# En bloc resection and reconstruction of a huge chondrosarcoma involving multilevel upper thoracic spine and chest wall: case report

**DOI:** 10.1186/s12891-021-04208-6

**Published:** 2021-04-12

**Authors:** Xiaodong Tang, Zhenyu Cai, Ruifeng Wang, Tao Ji, Wei Guo

**Affiliations:** Musculoskeletal Tumor Center, Peking University People’s Hospital, No. 11 Xizhimen South Street, Xicheng District, Beijing, 100044 China

**Keywords:** Upper thoracic spine, Chondrosarcoma, En bloc spondylectomy, Chest wall, Case report

## Abstract

**Background:**

En bloc resection of malignant tumors involving upper thoracic spine is technically difficult. We surgically treated a patient with grade 2 chondrosarcoma involving T1–5, left upper thoracic cavity, and chest wall.

**Case presentation:**

A 37 years old, male patient was referred to our hospital for a huge lump involved left shoulder and chest wall. In order to achieve satisfied surgical margins, anterior approach, posterior approach, and lateral approach were carried out sequentially. After en bloc tumor resection, the upper thoracic spine was reconstructed with a 3D-printed modular vertebral prosthesis, and the huge chest wall defect was repaired by a methyl methacrylate layer between 2 pieces of polypropylene mesh. Postoperatively, the patient suffered from pneumonia and neurological deterioration which fully recovered eventfully. At 24 months after operation, the vertebral prosthesis and internal fixation were intact; there was no tumor local recurrence, and the patient was alive with stable pulmonary metastases.

**Conclusion:**

This case report describes resection of a huge chondrosarcoma involving not only multilevel upper thoracic spine, but also entire left upper thoracic cavity and chest wall. Although with complications, en bloc tumor resection with combined surgical approach and effective reconstructions could improve oncologic and functional prognosis in carefully selected spinal tumor patients.

## Background

En bloc resection has been proven as an effective treatment for aggressive benign, primary malignant, and solitary metastatic tumors involving lumbar and thoracic spine [1–3]. Satisfied tumor local control can be expected in patients with adequate surgical margins. However, en bloc resection for large size tumor invading multilevel vertebrae, especially in upper thoracic spine is more complicated and has a higher complication rate compared with single level resection [4, 5]. For only few cases have been reported, it’s worthwhile to have more experience on this issue.

Here, we present a case of chondrosarcoma involving T1–5, left upper thoracic cavity, and chest wall. The details of surgical approaches, technique of en bloc resection, reconstruction of spine column, and repair of thoracic cavity have been described thoroughly.

## Case presentation

**Fig. 1:**
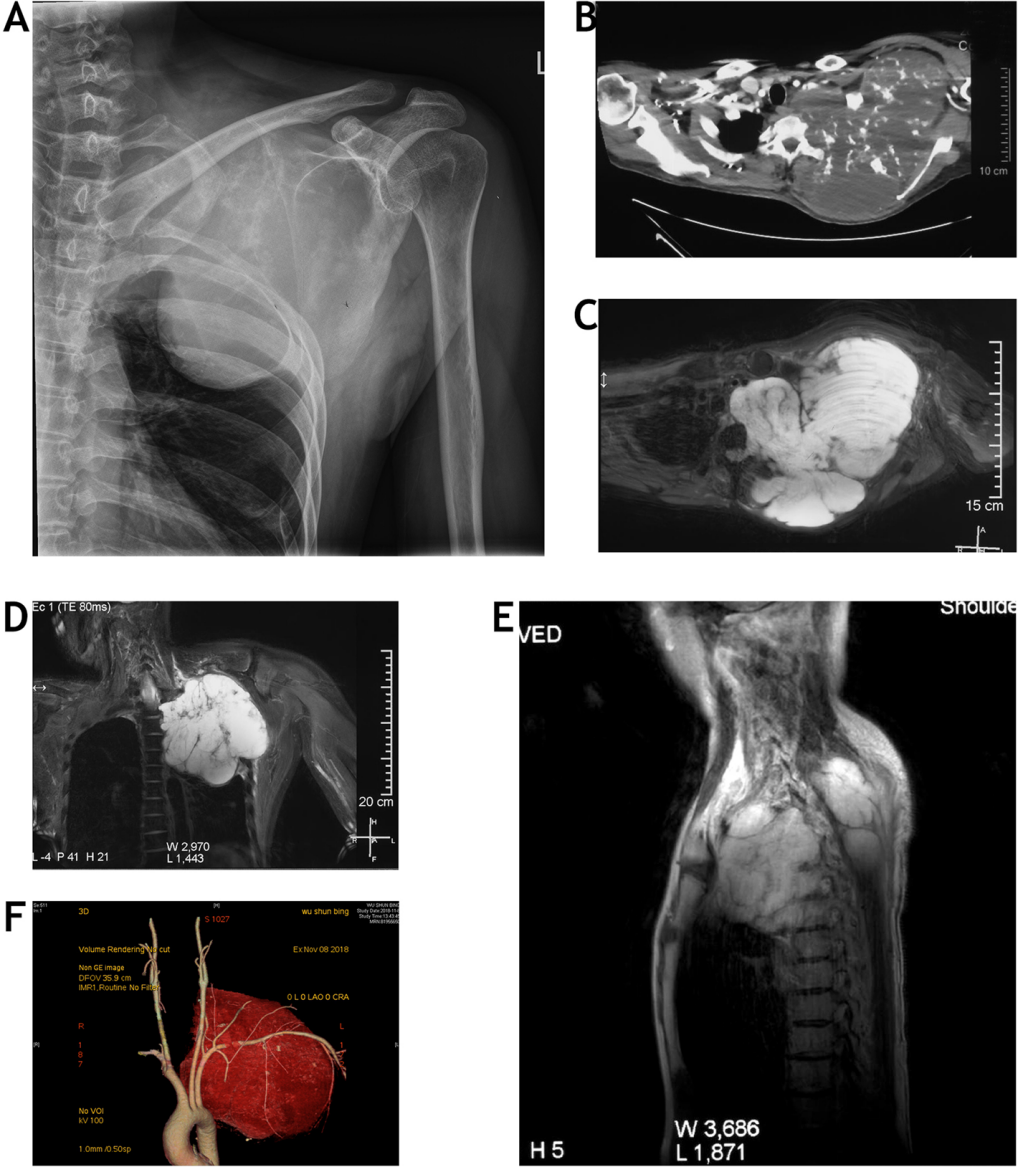
The preoperative plain radiograph (**a**), enhanced computed tomography (**b**), T2-weighted axial view (**c**), coronal view (**d**), and sagittal view (**e**) images show tumor involvement of T1-T5 and left thoracic cavity. The left subclavian artery is compressed by tumor on three-dimensional image reconstruction (**f**)

A 37 years old, male patient was referred to our hospital for a huge lump involved left shoulder and chest wall. The patient was suffered from severe radiating pain in the left upper extremity for 1 year. Physical examination showed decreasing sensation on the ulnar side of left hand, normal muscle strength in the upper extremity, and normal muscle strength and sensation in the lower extremity (Frankel D). The preoperative imaging findings (Fig. 1a-e) showed a huge tumor mass (19 × 16 × 17 cm) involving T1-T5, left thoracic cavity, and wall of the chest. The tumor displaced the contents of mediastinum right sided and ventrally, and suspiciously invaded left subclavian artery (Fig. 1f). The pathological diagnosis of grade 2 chondrosarcoma was confirmed by core needle biopsy. Preoperatively, the treatment protocol was discussed thoroughly by a multi-disciplinary team including orthopaedic oncology surgeons, vascular surgeons, thoracic surgeons, and medical oncologists. The surgical plan to achieve en bloc resection was made according to Weinstein-Boriani-Biagini (WBB) surgical system [6]. Because the tumor was particularly huge, a modified strategy of type 4 resection proposed by Boriani et al. [7] was adopted, which means the tumor will be removed via an anterior approach. Preoperative superselective endovascular embolization was carried out for this patient 1 day before the operation.

## Surgical procedure

### Stage I: anterior approach

**Fig. 2:**
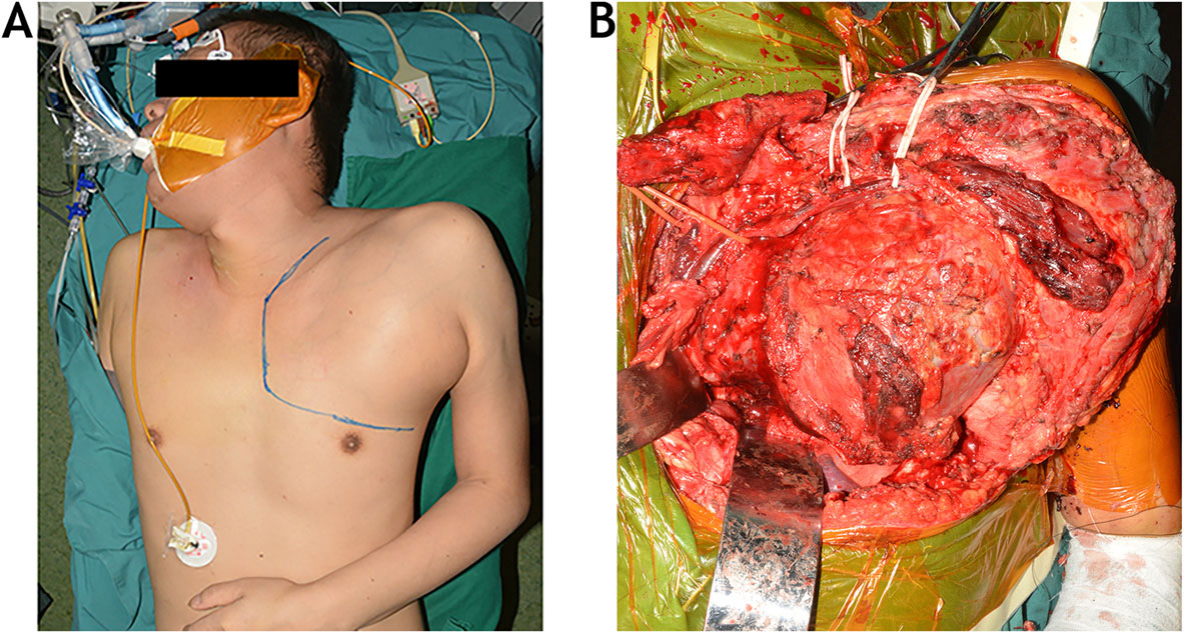
During the operation, an anterior “trap door” approach (**a**) was adopted in the first stage. The anterior aspect of tumor was exposed after isolation of the main arteries, veins, trachea, esophagus, and superior lobe of left lung (**b**)

The patient was placed in the supine position, and a longitudinal roll was situated along the spine between the scapulas. The head was gently rotated toward the right. After preparing and draping the patient in the usual sterile fashion, a “trap door” exposure [8] was carried out (Fig. 2). The initial incision was made over the anterior part of the left side of the neck, which was approximately 2 cm proximal and parallel to the left clavicle. A vertical limb beginning at the medial end of the transverse incision extended distally along the midline of sternum to the third interspace and out along the fourth interspace to the anterior axillary line. The subcutaneous tissue and platysma were cut, and the resulting two large flaps were retracted to provide a broad exposure of the sternal manubrium, the clavicle and sternocleidomastoid muscle, and the entire supraclavicular fossa. The pectoralis major muscle was disinserted from the medial lower half of the clavicle and from the upper part of the sternum to reveal the costosternal joint. The sternoclavicular joint capsule was spared. Osteotomy of the clavicle was performed after a small amount of periosteum from the middle third of the clavicle was removed. The costosternal cartilage of the first rib attached to the sternum was resected. The medial half of the clavicle and the left upper outside angle of the sternum were elevated toward the cranium after an L-shaped osteotomy was made on the manubrium. The left part of sternum and the anterior part of second, third, and fourth rib were rongeured to enhance the exposure of anterior mediastinum and left thoracic cavity. The fourth interspace is entered to the anterior axillary line. In the cephalad edge of tumor, the main arteries (the arch of aorta, left common carotid artery, and left subclavian artery) and veins (the left brachiocephalic and subclavian vein) were identified and isolated. After scalene muscles were released from the first rib, the C8 and T1 nerve roots invaded by tumor were transected. The trachea and esophagus were retracted medially. The superior lobe of the left lung was separated from the caudal edge of tumor. After fully visualizing the anterior aspects of the C7 through T6 vertebral bodies, the intervertebral discs of C7–T1 and T5–6 were rongeured. A silicon sheet was inserted between the tumor and the anterior mediastinum for better identification of the tumor margin from a posterior approach. The osteomuscular flap of manubrium and medial clavicle were temporary reduced, and the wound was then closed.

### Stage II posterior approach

**Fig. 3:**
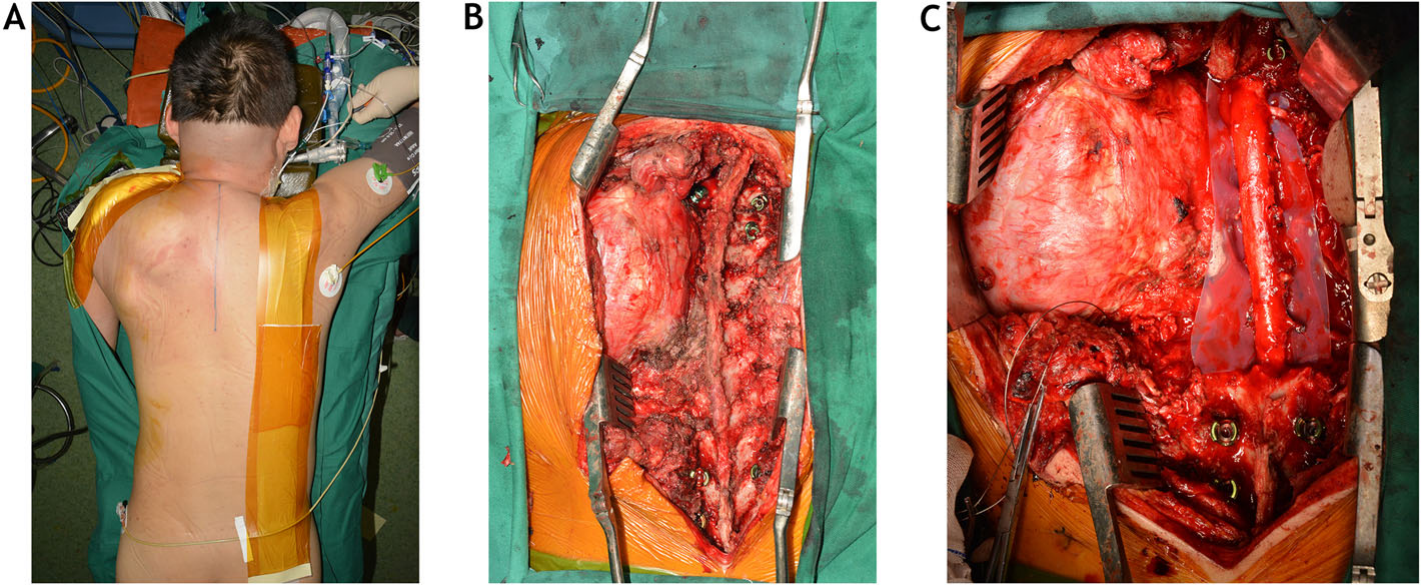
In the second stage, posterior midline incision (**a**) was made to expose posterior aspect of the tumor (**b**). After laminectomy, a silicon sheet was inserted between tumor involved vertebrae and dural sac (**c**)

The patient was positioned prone on the operating Table. A posterior midline incision (Fig. 3) was made from C4 to T8. After the exposure was completed, instrumentations were bilaterally placed into the lateral masses of C4–6 and pedicles of T6–8. On the right side, after the resection of posterior ribs and costotransverse joints from T1 through T6, the T2–5 nerve roots and T1–5 segmental vessels were ligated and transected. A blunt dissection was then performed between vertebral bodies and pleura to reach the anterior longitudinal ligament. On the left side, the trapezius and the rhomboid muscles were elevated and retracted laterally, allowing mobilization of the left scapula and exposing the posterior chest wall from the T1 through T6 ribs. A transverse incision starting from the posterior midline was adopted along the fourth interspace and extended laterally to the posterior axillary line. The rib heads of T5 and T6 were resected to allow entrance into the left thoracic cavity. After laminectomies were performed from C7 through T6, the left C8–T5 and the right T2–5 nerve roots were ligated and sectioned in the spinal canal. The C7–T1 disc and posterior longitudinal ligament were bilaterally revealed between C8 and T1 nerve roots, and resected as much as possible. At the T5–6 level, a Tomita saw was lassoed around the vertebral body while protecting the anterior great vessels and posterior dural sac. The two ends of Tomita saw were sutured with surrounding tissue on the left side of the T5–6 disc space. A tapered rod measuring 3.5–6.0-mm was placed between C4 and T8 on the right side for temporary fixation. A silicon sheet was inserted between vertebrae and dural sac, and the wound was then closed. The patient was sent back to intensive care unit, and next stage operation was going to be started 2 days later.

### Stage III lateral approach

**Fig. 4:**
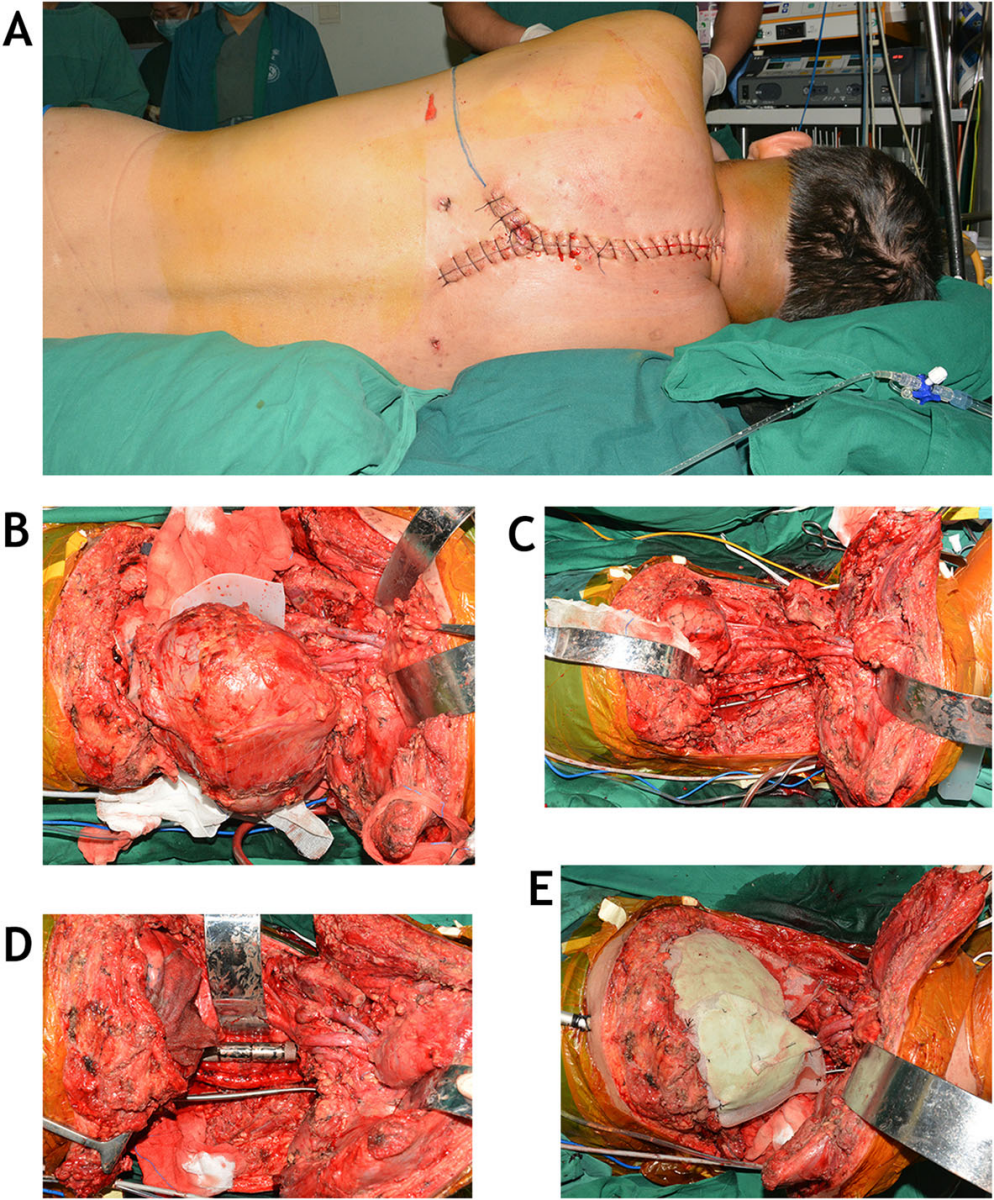
In the third stage, a parascapular thoracotomy connected previously anterior and posterior incisions (**a**). After elevated the scapular, the whole tumor mass was revealed from lateral approach (**b**). Once the tumor was removed (**c**), the anterior column between C7 and T6 was reconstructed with a 3D-printed modular vertebral prosthesis (**d**). Finally, a methylmethacrylate layer sandwiched between 2 pieces of polypropylene mesh was used for the chest wall reconstruction (**e**)

The patient was repositioned in the right lateral decubitus position. The upper left extremity was sterilized and draped on the operating Table. A single transverse incision connecting the ventral and dorsal sites was made on the fourth rib interspace (Fig. 4). The previous anterior and posterior incisions were then reopened. After detaching the serratus anterior muscle, the scapula and arm were lifted toward the cephalad direction to show the left chest wall. A thoracotomy was performed through the left fourth rib space. The left side of T5–6 vertebrae was exposed, and the osteotomy through the T5–6 disc was completed by using the Tomita saw. The T1–5 vertebral bodies and the whole tumor mass were pushed ventrally and laterally. The residual disc and annulus at C7–T1 level were removed, and the specimen was delivered out of the left chest as a whole piece. In the posterior incision, another tapered rod measuring 3.5–6.0 mm was placed between C4 and T8 on the left side. The length of the resected vertebral bodies was measured, and a 3D-printed modular vertebral prosthesis (AK MEDICAL HOLDINGS LIMITED, Beijing, China) was assembled. The prosthesis was filled with granular bone allografts and placed from C7 to T6 for the anterior reconstruction and arthrodesis. Compression was placed on the prosthesis by compressing on the posterior rod-screw instruments. The broken lung tissue was repaired by a thoracic surgeon. A methyl methacrylate layer between 2 pieces of polypropylene (Marlex, Textile Development Associates, Inc., Franklin Square, NY) mesh was used for the chest wall reconstruction and to prevent the compression of the spinal cord by the lungs. The reduction of sternum and clavicle were performed using lag screws of cancellous bone and a dynamic compression plate, respectively. The wound was closed in a multilayered fashion.

## Postoperative course

**Fig. 5:**
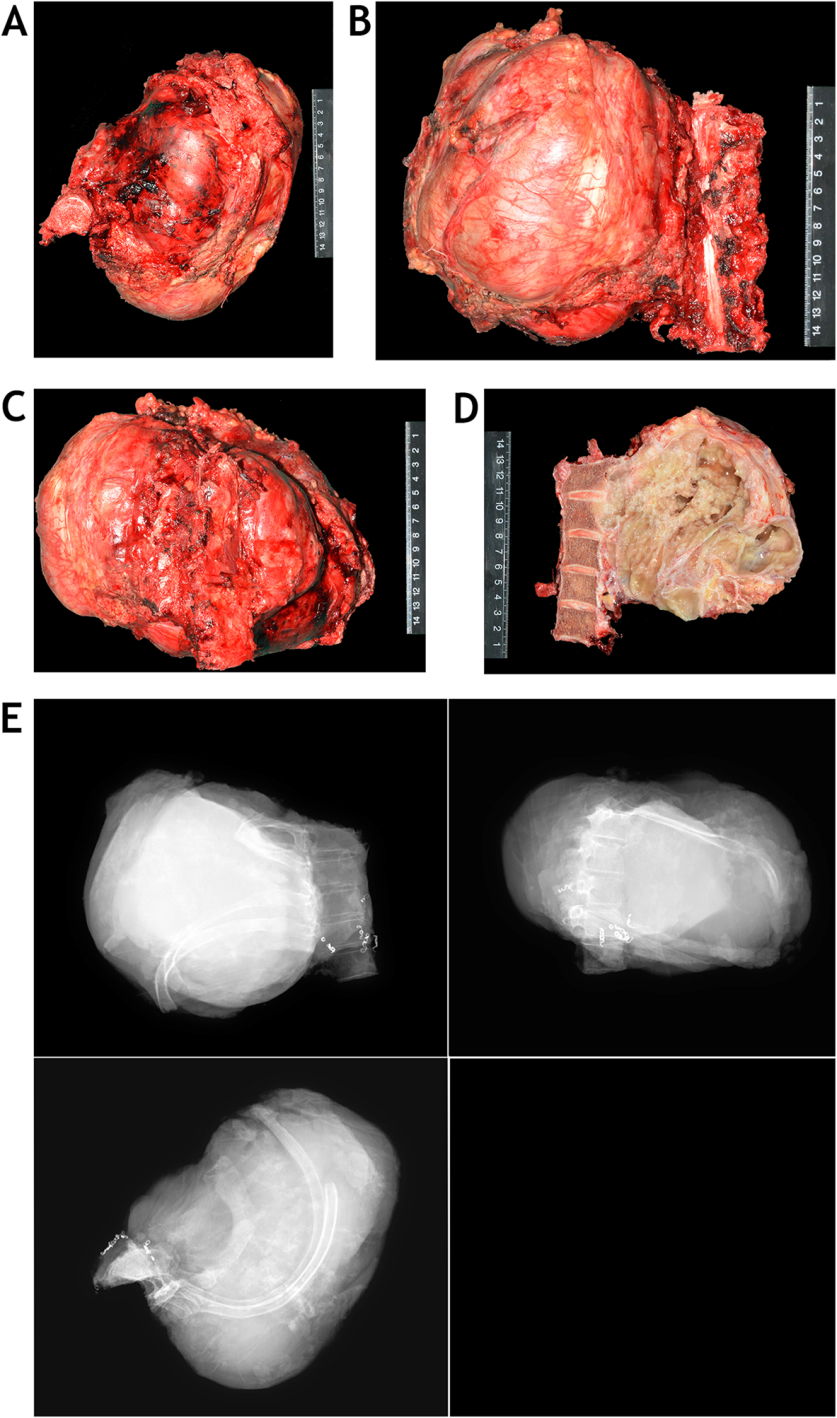
The gross view (**a**-**d**) and plain radiograph (**e**) of the specimen show en bloc resection of T1-T5 vertebras and left chest wall

**Fig. 6:**
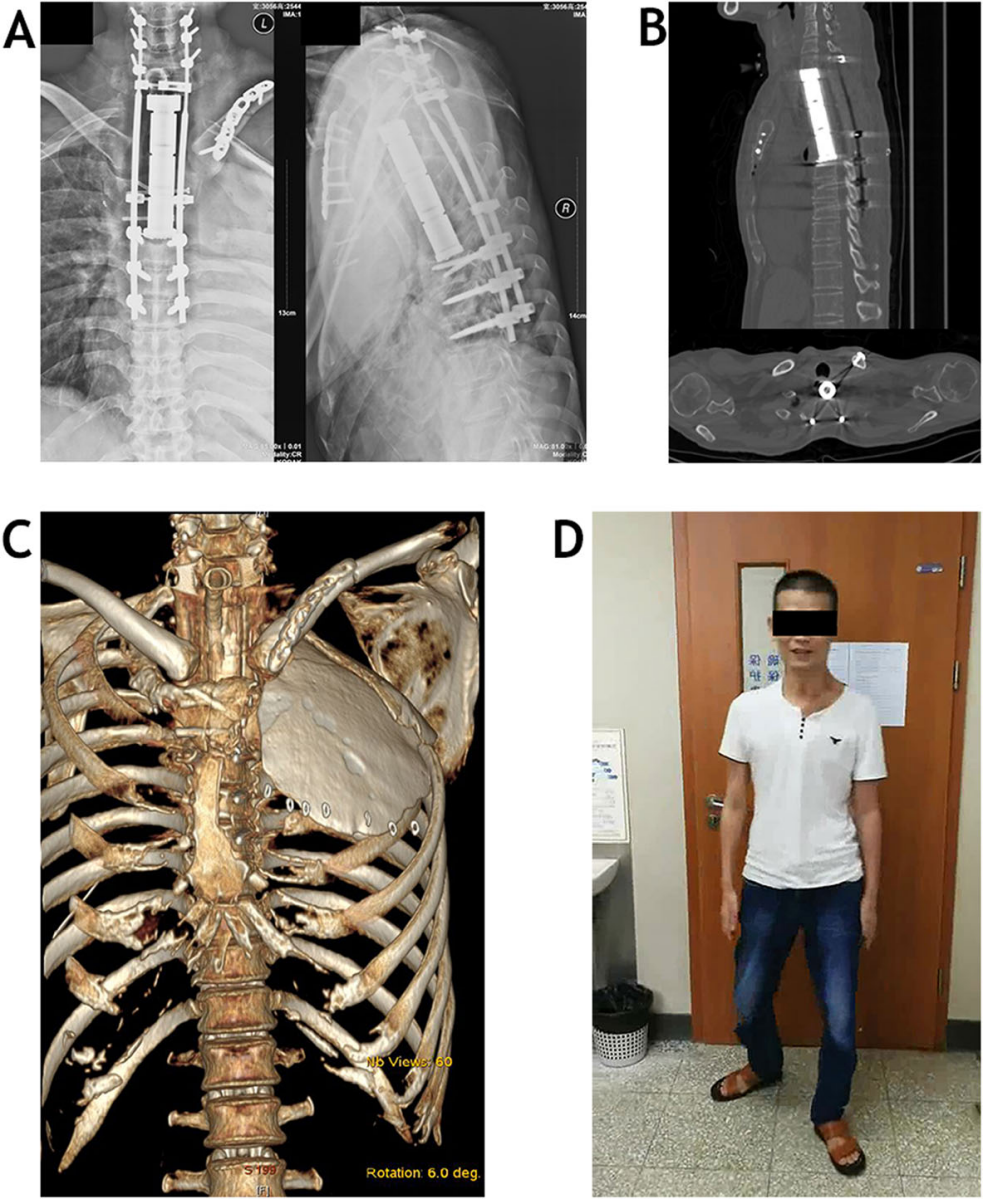
Postoperative plain radiograph (**a**), sagittal and axial view of computed tomography (**b**) show spinal reconstruction and internal fixation of sternum and left clavicle. The sandwiched bone cement plate for chest wall repair displays on Three-dimensional computed tomography reconstructions (**c**). This patient who walked independently was followed up at clinic 6 months after operation (**d**)

A nearly 3 kg tumor mass (Fig. 5) was completely removed after operations lasting for 26 h, with a 12.6 L blood loss volume. The surgical margins of specimen were evaluated both grossly and microscopically, and a marginal margin was achieved. The reconstructions of upper thoracic spine and chest wall were shown on postoperative radiographs (Fig. 6a-c). A chest tube was placed at the time of operation and chest drainage sustained 17 days. During perioperative period, the patient was suffered from pneumonia, and successfully treated with antibiotics for 3 weeks. This patient also went through postoperative neurological deterioration (Grade 2 power in bilateral lower limbs) which fully recovered to Frankel D at 6 months after operation (Fig. 6d). Multiple small nodules were found on pulmonary computed tomography at 8 months postoperative follow-up, and confirmed as metastases of chondrosarcoma by thoracoscopy. Then, the patient received targeted therapies (Apatinib 250 mg orally once daily with regorafenib 80 mg 2 weeks’ on 1 week off) according to the suggestions of medical oncologist. At latest 24 months postoperative follow up, the vertebral prosthesis and internal fixation were intact; there was no tumor local recurrence, and the patient was alive with stable disease.

## Discussion

TES which provides the best chance of local and system control for primary spinal malignant tumors has been developed for many years. With the help of modern techniques, wide surgical margin could be expected in some patients receiving spinal tumor surgery. In a review [1] of 103 patients affected by primary spinal tumors, the rate of achieving adequate margins was 82.4%, whereas local recurrence occurrence rate was 21.4% (22 patients). Marginal and intralesional resections were independent predictors for local recurrence and tumor-related mortality (15.5%). When tumors involve more than one vertebra, multilevel TES can provide satisfactory oncological prognosis. In a study [9] including 20 patients who received multilevel (2–5 segments) en bloc spondylectomy of the thoracolumbar spine, wide and marginal surgical margins were achieved in all patients. With a mean follow-up of 25.0 months, one patient had local recurrence, whereas two patients died of systemic disease. In another study [10] of multilevel spondylectomy for spine tumors, the local recurrence rate was 8, and 81% of patients survived without evidence of the disease at the final follow-up.

In order to achieve satisfied surgical margins for spinal tumor resection, different approaches are recommended according to the scope of tumor invasion. Boriani et al. [7] have proposed four kinds of approaches (types) for en bloc resection of thoracic spine tumors: anterior approach, posterior approach, anterior approach first and posterior approach second, and posterior first and simultaneous anterior and posterior approach second. Among them, Type 4 resection is more suitable when the tumor is particularly huge and it is presumed that the resection can require complex maneuvers. Therefore, combined anterior and posterior approach should be considered in this patient. Because the upper thoracic spine remains an extremely difficult region to approach anteriorly (proximity of the mediastinal contents, the heart, and the great vessels), different approaches to the cervical thoracic junction including low cervical approach combined with sternotomy [11], unilateral or bilateral manubriotomy [12], or with clavicular dissection [13] have been reported. The anterior exposure of tumor with large diameter involving upper thoracic spine could be even more complex, and additional lateral parascapular thoracotomy may be considered. Siubba et al. [14] reported of a 5-level spondylectomy (T1–5) for en bloc resection of an extensive thoracic chordoma via a bilateral thoractomy. In our case, for the tumor which involved left thoracic cavity and chest wall had even bigger volume when compared with Siubba’s, a “Trap door” incision combined with left parascapular thoracotomy were adopted for better anterior exposure.

With aggressive multilevel TES, a large spinal column defect is created and needs effective anterior spinal column reconstructions. Widely used anterior reconstruction techniques include vascularized autograft strut, nonvascularized autograft strut, titanium cage, stackable carbon cage system, structural allograft strut, and PMMA [15]. All these methods have advantages and deficiencies. For example, mesh cages filled with granular bone grafts have a high incidence of subsidence; the expandable cage has the disadvantages of insufficient amount of bone graft and limitation of using in extensive spinal column reconstruction; vascularized autograft has shortcomings of donor site morbidity and time consumption of vascular anastomosis. Therefore, a 3D-printed modular vertebral prosthesis assembled during operation was adopted for this patient. The prosthesis has variable combinations of length, diameter, and degree of curvature to fit different needs of vertebral reconstruction.

In addition to spinal defect, this patient had also faced to the problem of large area of chest wall reconstruction. For patients with multi-level thoracic spinal tumors involving huge rib cage, large area of chest wall defects could cause severe respiratory complications, such as paradoxical chest wall movement and respiratory failure [16]. Several methods for rib cage reconstruction during operation of TES have been reported in few studies with optimal results. Czyz et al. [17] used rib plate combined with flexible polyethylene terephthalate band for chest wall reconstruction in a case of Pancoast tumor resection. Xiao et al. [18] reported five patients of multilevel TES of thoracic spine combined with large area of ribs and chest wall resection. Titanium rods used for rib reconstruction were modified accordingly to attach to the screw-rod system proximally, and the distal end of rods was dynamically inserted into the ribs. However, for the concern of complications [16] including wound infection and hardware failure, and difficulty of full length of rib rebuilding, a sandwiched bone cement plate [19] with curvature was adopted for chest wall reconstruction in our patient. It’s easily maneuvered during operation, with no complications, and allowed excellent recovery of pulmonary function.

We successfully resected a huge chondrosarcoma involving T1–5, left upper thoracic cavity, and chest wall as one piece, and the patient fully recovered his neurologic compromise eventually. Although encountered complications, en bloc tumor resection with combined surgical approach and effective reconstructions could improve oncologic and functional prognosis in carefully selected spinal tumor patients.

## Data Availability

The datasets used and/or analysed during the current study available from the corresponding author on reasonable request.

## Abbreviations

TES: Total en bloc spondylectomy
WBB: Weinstein-Boriani-Biagini
